# A Case of Imatinib-Induced Hepatitis

**DOI:** 10.7759/cureus.1302

**Published:** 2017-06-01

**Authors:** Osman Bhatty, Mohammad Selim, Thamer Kassim, Lakshmi Chintalacheruvu, Manuel Urra, Sonia Shah, Joseph Haggerty, John Gross, Aravdeep Jhand, Gene Pershwitz, Jaya Gupta

**Affiliations:** 1 Internal Medicine, Creighton University; 2 Internal Medicine, Creighton University School of Medicine; 3 Internal Medicine, CHI Creighton University Medical Center; 4 Pathology, Creighton University School of Medicine

**Keywords:** imatinib, gleevec, chronic myeloid leukemia, drug-induced hepatitis

## Abstract

A 71-year-old female with a past medical history of Philadelphia chromosome-positive chronic myelogenous leukemia on imatinib therapy, Sjogren’s syndrome, and hypothyroidism presents with acute hepatitis. After a comprehensive workup ruling out viral, infectious and metabolic etiologies imatinib is stopped which results in immediate improvement. The biopsy is consistent with drug-induced liver damage; the patient is started on oral prednisone and discharged. Unfortunately, our patient’s liver function does not improve over the course of the next week and she is readmitted for hepatic and renal failure. During this second admission patient’s condition continues to deteriorate with concomitant gastric bleeding, renal injury, and cellulitis. She ultimately chooses a palliative approach.

## Introduction

Imatinib is a specific tyrosine kinase inhibitor that has been successful in treating Philadelphia chromosome-positive chronic myelogenous leukemia (CML) as well as gastrointestinal stromal tumors (GIST). Both these diseases are marked by constitutively expressed tyrosine kinase that causes unregulated cell growth and proliferation. Imatinib inhibits the unique BCR-ABL transfiguration that is a result of a translocation between chromosomes 9 and 22. Imatinib was the first antineoplastic drug that specifically was directed at a molecular abnormality. Inhibition of this enzyme leads to reversal of progression and ultimately is highly effective. A similar enzyme abnormality was found in GIST. This drug was approved in the US in 2001 and had full approval in 2005 for the indication of Philadelphia chromosome positive CML or acute lymphoblastic leukemia and unresectable or metastatic GIST with positive kit. The typical doses ranged from 400 to 600 mg once a day [1].

## Case presentation

**Figure 1:**
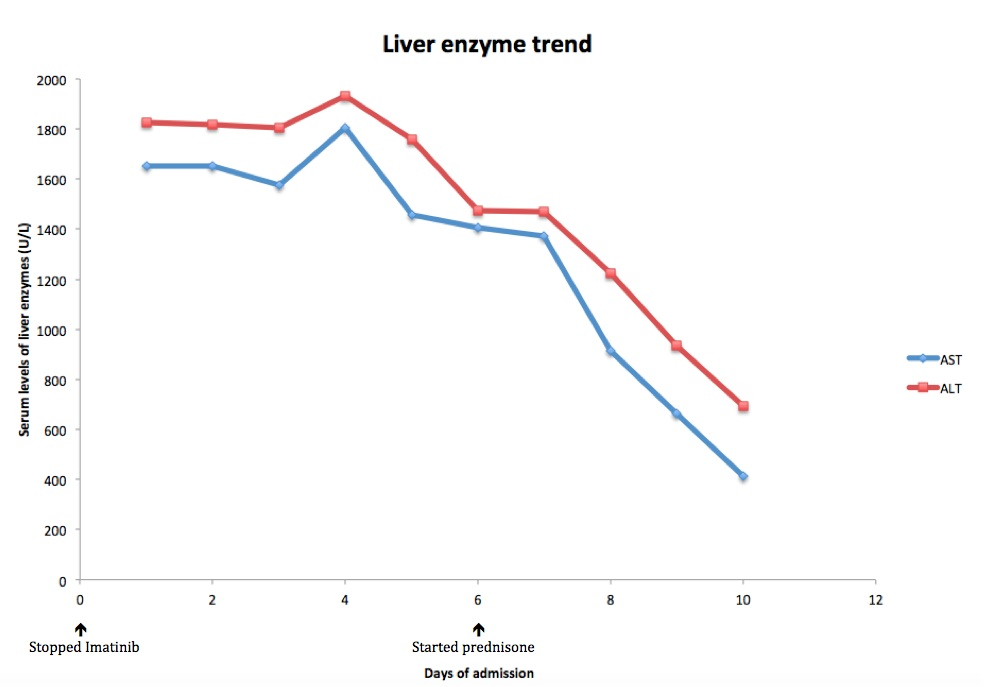
Liver enzyme trend. Hepatic enzyme trend through admission and after starting steroid therapy.

**Figure 2:**
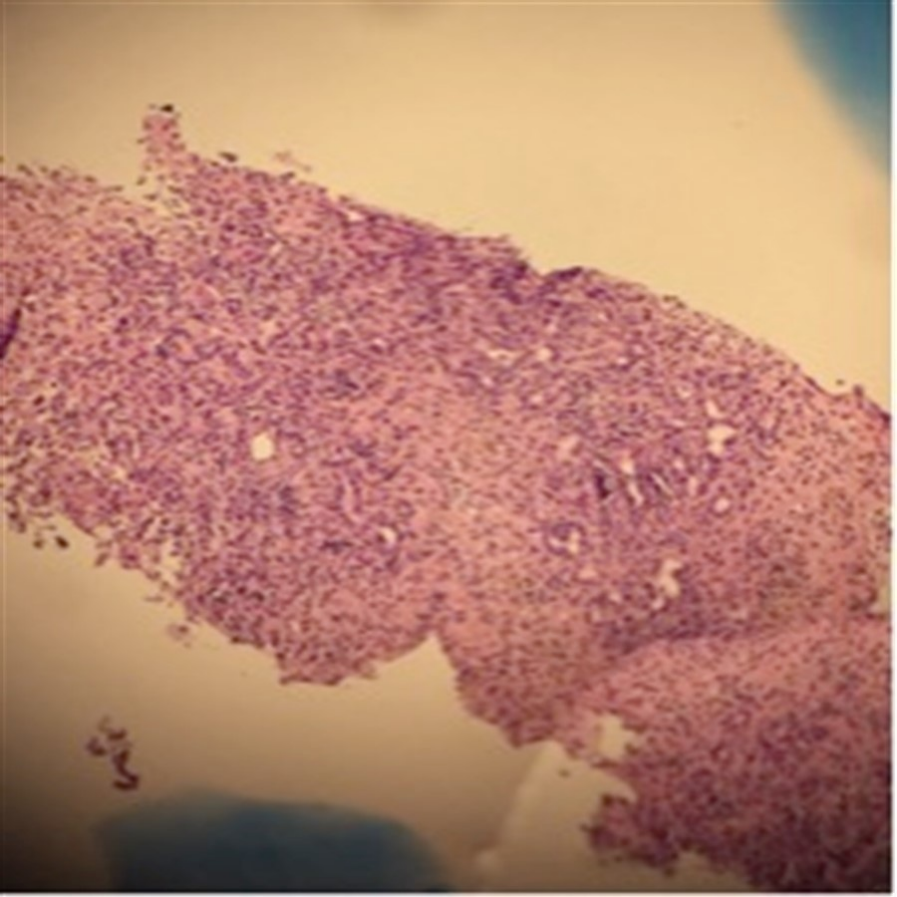
Spotty necrosis of hepatic lobular unit with adjacent lobule relatively preserved.

**Figure 3:**
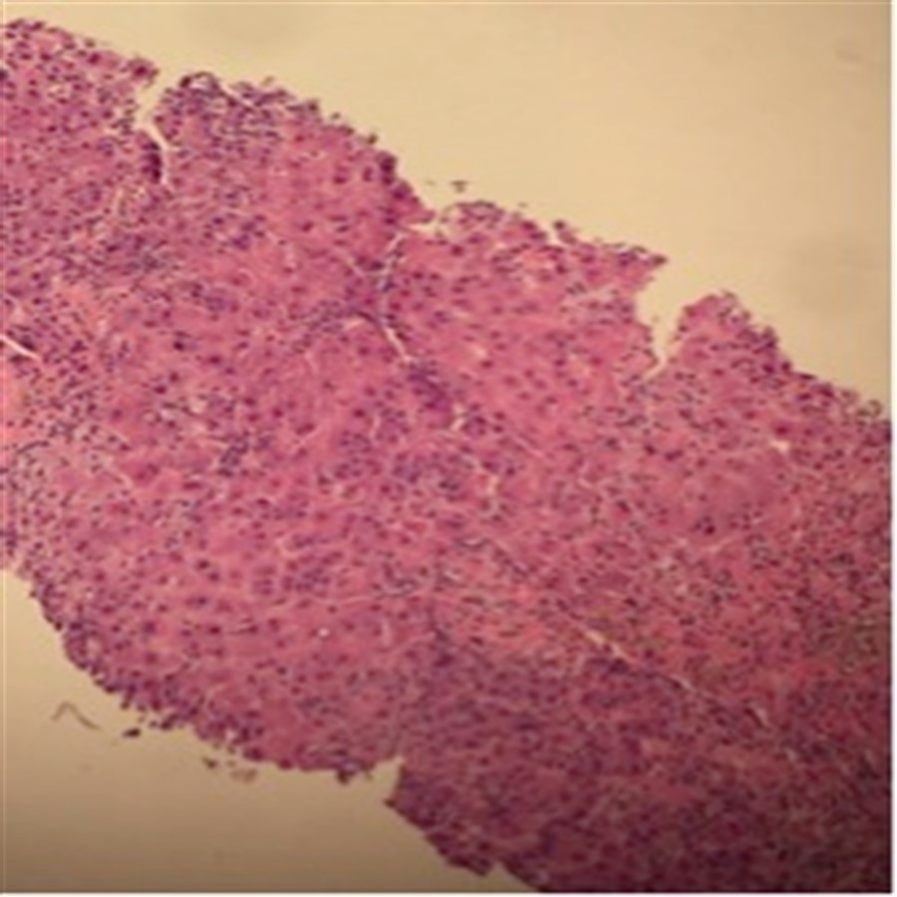
High power view of acute hepatitis and acute cholangitis with hepatocyte necrosis, cholestasis and neutrophilic infiltration of bile ductules.

**Figure 4:**
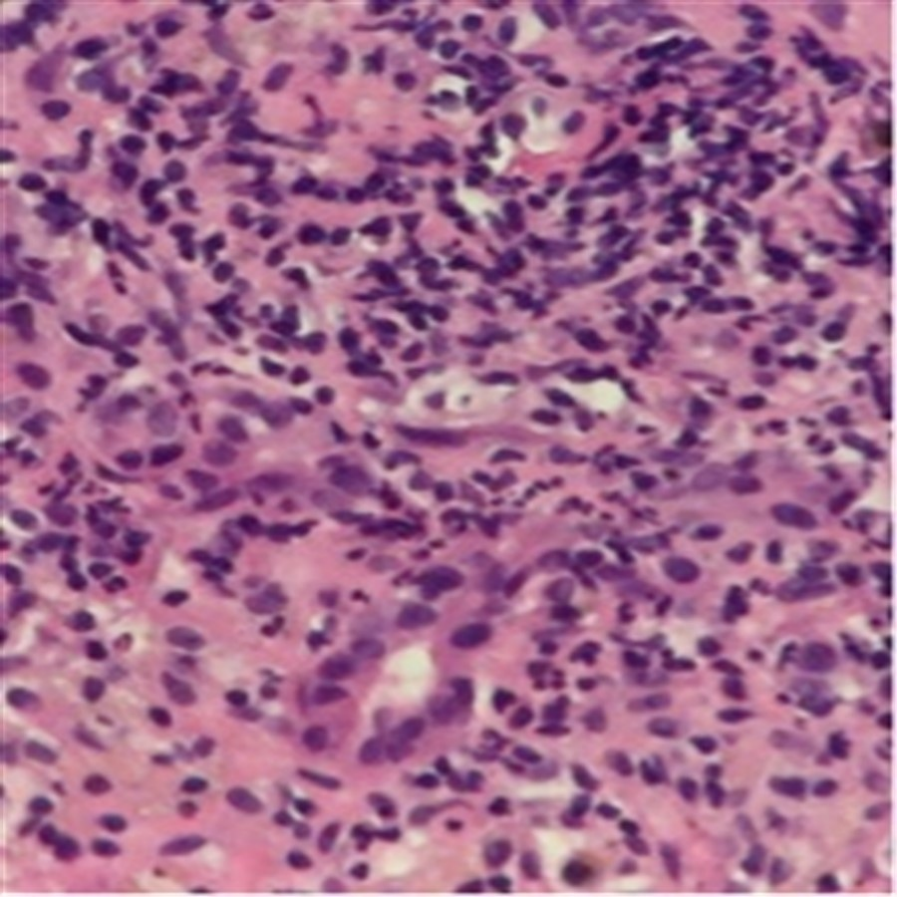
Medium power view of acute hepatitis with cholestasis. Notably, severe bile duct damage and ductopenia is identified.

**Figure 5:**
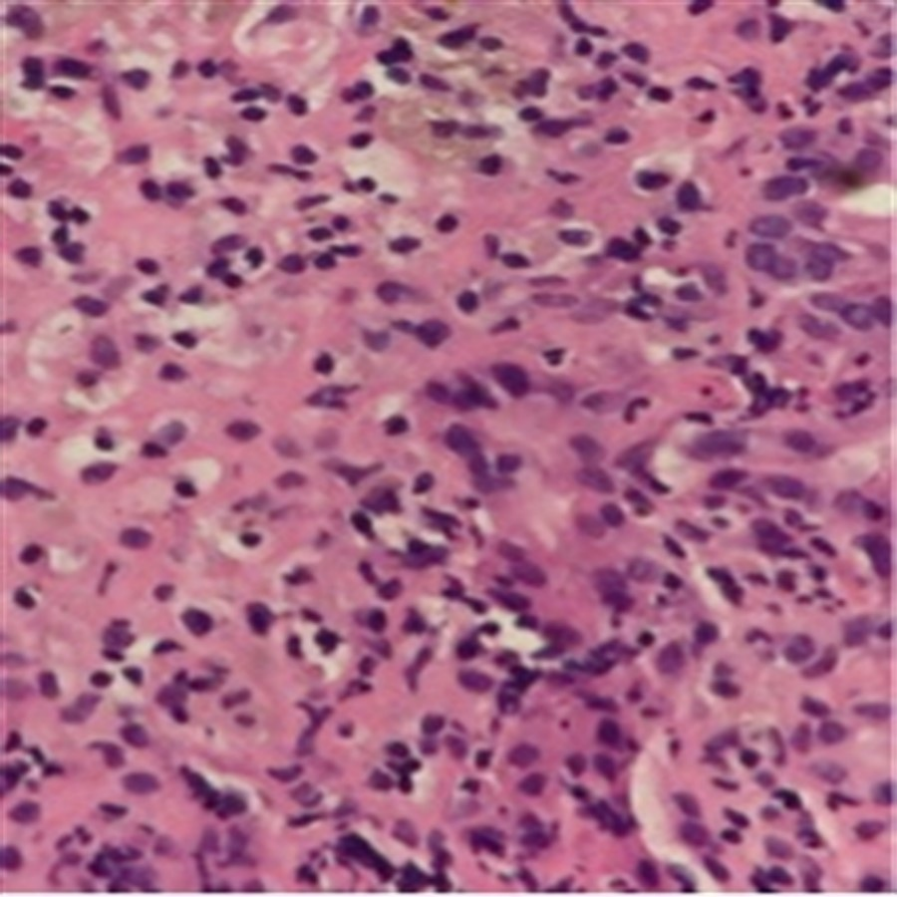
High power view of acute hepatitis (neutrophilic infiltration of hepatic parenchyma with early coagulative necrosis of hepatocytes).

A 71-year-old Caucasian female with past medical history significant for CML and Sjogren’s syndrome presented with new onset of jaundice first noticed by her daughter two days prior. At presentation, her only complaint was that of generalized weakness. Initial workup revealed markedly elevated transaminases with aspartate aminotransferase (AST) of 1650 IU/L and alanine aminotransferase (ALT) of 1816 IU/L along with an increased total bilirubin of 14.7 mg/dL and direct bilirubin of 11.9 mg/dL. Alkaline phosphatase was borderline high at 132 IU/L and her international normalized ratio (INR) was elevated at 2.1. Her model for end stage liver disease (MELD) score was 28 on arrival. Upon review of her medications she had been started on Imatinib six months prior for her CML, this medication was held on admission due to possible hepatotoxicity. Prior, liver function tests (LFTs) taken in-between this period were all within normal limits. Acetaminophen level on arrival was negative. Given her history of Sjogren’s and her acute presentation, an autoimmune workup was performed that included antinuclear antibodies (ANA), anti-smooth, anti-mitochondrial, and anti-thyroid peroxidase (TPO) serology, all of which came back negative. Immunoglobulin IgM and IgG were both slightly elevated. Infectious workup for cytomegalovirus (CMV), Epstein-Barr virus (EBV), hepatitis c virus (HCV), and hepatitis B virus (HBV) were all negative. An abdominal ultrasound revealed a large calculus in the gallbladder but no obstructive process was noted. After reversal of INR with fresh frozen plasma (FFP) the patient underwent an interventional radiology (IR) guided liver biopsy. Preliminary results showed findings consistent with drug-induced liver damage. The patient was started on prednisone 20 mg, two days later her transaminases decreased (Figure 1) significantly with an AST of 914 IU/L and ALT of 1222 IU/L. The transaminases continued to trend down reaching AST of 412 IU/L and ALT of 692 IU/dL on the day of discharge. Bilirubin peaked on day 6 of admission and remained stable at 20 mg/dL for the rest of her hospital stay. INR was 2.0 at the time of discharge after three days of Vitamin K administration. Final pathology report noted acute cholangitis and hepatitis consistent with drug-induced liver injury (Figures 2-5). The patient was sent home on 20 mg prednisone for one month with an eventual taper down to 5 mg before being discontinuing.

 

The patient was readmitted two weeks later with progressive weakness, fatigue, and abdominal pain. On physical examination, right upper quadrant tenderness and bilateral lower extremity edema were noted. Labs were significant for an elevated white blood cell count of 20.0 × 109/L, red blood cell count and platelets at baseline (2.9 M/uL and 122 K/uL, respectively) and elevated serum creatinine of 1.5 mg/dL. Liver function tests were as follows: AST 145, ALT 200, ALP 239, and total bilirubin of 22.0. Due to concerns for acute cholangitis, an endoscopic retrograde cholangiopancreatography (ERCP) procedure was performed. No biliary dilatation was observed; however, a 2 cm incidental gastric ulcer was seen with a visible vessel and fresh adherent clot in the pre-pyloric region. Cauterization of the ulcer was performed. Testing for H.pylori was negative. Prednisone was discontinued in the setting of high risk for gastrointestinal (GI) bleed and the patient was initiated on intravenous (IV) proton-pump inhibitor.

Over the next few days, the patient’s white blood cell count continued to rise and she developed a petechial rash over her lower extremities. She was treated for presumed cellulitis with parenteral antibiotics. Her kidney function also continued to worsen and was attributed to nonsteroidal anti-inflammatory drug (NSAID)-induced acute tubular necrosis. The patient started experiencing melena with acute drop in her hemoglobin requiring packed red cell transfusions. A repeat esophagogastroduodenoscopy was performed which revealed “oozing” but no active bleeding from the previously identified ulcer. Thus, no intervention could be performed. Prognosis in the setting of acute hepatic and renal failure with concomitant GI bleed was very poor, hence the patient decided to withhold further treatment and expressed desire to pursue hospice care. The patient eventually expired 24 days after the second admission.

## Discussion

Elevations in serum aminotransferase levels are common during therapy but ALT levels greater than five times the upper limit of normal occur less frequently at around 2-4% [1-2]. There are several case reports in the literature linking clinical acute liver injury with jaundice to imatinib therapy that range from 6 days of starting to several years [1]. The pattern of these diseases is usually hepatocellular, though cholestatic pictures like in our patient have also been reported. Data surrounding therapy of these drugs is scarce but discontinuation of the drug and immunosuppression via prednisone has been shown to be effective. There have been several instances of reactivation of chronic hepatitis B during therapy as well, which presents similarly to an acute hepatitis-like syndrome [1]. Some authors report that only five cases of imatinib-induced severe acute hepatitis have been reported all of which improved with interruption of the drug [3]. Mechanisms of injury, however, are not well known at this time – hypersensitivity has been suggested as well as the production of metabolites due to the drug’s metabolism through the CYP3A4 system [3]. This is a point of interest since drug-related autoimmune injuries are underreported due to difficulty in differentiating between these and drug-related injuries. One case report has been documented surrounding imatinib liver injury with autoimmune features that our own case shares features with.

The relationship between drug-induced liver injury (DILI) and autoimmune hepatitis (AIH) is complex and Weiler-Normann in 2011 established several classifications [4]. First, AIH with DILI is essentially reactivation of a prior known AIH though it is often difficult to prove a causal relationship. Second, drug-induced AIH occurs in patients with no prior history of AIH; this is essentially an immune reaction in a genetically predisposed individual which results in a permanent need for immunosuppression. Third, immune-mediated DILI which resolves with removal of the offending drug and is often seen in culprits such as nitrofurantoin or minocycline and can present as a mixed type of liver damage in which immunosuppression is mandatory and life-saving. The fourth type suggested by Castiella, et al. in 2014 is a mixed autoimmune type which presents with features of drug-induced autoimmune hepatitis (DI-AIH) and immune-mediated drug-induced live injury (IM-DILI). Finally, a subset of patients that present with DILI and positive autoantibodies, as in 2002, Ohmoto and Yamamoto studied patients admitted with DILI and identified six with positive ANA that may have also had autoimmune disease, and suggested these should be followed over the long term even if liver function recovered [4].

Unfortunately, liver histology alone cannot differentiate between DILI and AIH which may suggest that any immune-mediated drug-induced liver disease is likely underreported [4]. As such the best way to diagnose individuals that present to the hospital with acute hepatitis with a drug possibly being a culprit is stopping treatment – if transaminases decrease more than 50% in one week to one month than DILI is likely the final diagnosis [4]. If enzymes are permanently raised or increased during follow-up and autoantibodies are positive than AIH is assumed [4], also if AIH scale reveals likely AIH, then treatment with immunosuppression should be initiated [4].

The mechanism of imatinib-induced liver injury is not fully understood. In 2005, Ferrero, et al. described five patients with CML on imatinib therapy at 400-600 mg/day, who after two to eight months of therapy had elevated to liver function test that was indicative of grade 3 to 4 liver injury per the WHO [5]. Liver biopsies were performed in all patients with necrosis, fibrous scars, and periportal inflammation. Of significance, all five were given corticosteroid therapy at low doses (25-37 mg/day) which resulted in unanimous normalization of liver enzymes in two to four weeks. Imatinib was then resumed without recurrence of injury and all five patients were able to finish treatment into cytological remission [5]. This is one of the few case reports the authors were able to find about treatments strategies for imatinib-induced liver injury.

## Conclusions

Imatinib can cause drug-induced liver injury and should be on the differential for any patient on treatment presenting with acute hepatitis. Discontinuation of this drug and improvement in liver enzymes is the key to diagnosis. Corticosteroids at low intermediate dosage look promising as a treatment modality in reversing imatinib-induced hepatotoxicity. There are few case reports pertaining to acute liver failure secondary to imatinib and even less discussing treatment failure when patients are on corticosteroids. Furthermore, this case is unique given findings of autoimmune hepatitis; readers should remember to obtain a liver biopsy when stopping the drug alone does not improve liver function tests or patient status. This case report aims to add to the literature surrounding a potentially devastating clinical consequence and to remind physicians to closely monitor liver enzymes when using this drug.
